# Brain training: rationale, methods, and pilot data for a specific visuomotor/visuospatial activity program to change progressive cognitive decline

**DOI:** 10.1002/brb3.196

**Published:** 2013-12-31

**Authors:** William J Tippett, Mireille N Rizkalla

**Affiliations:** 1 Department of Psychology, University of Northern British ColumbiaPrince George, British Columbia, Canada; 2 School of Health Sciences, University of Northern British ColumbiaPrince George, British Columbia, Canada; 3 Heart and Stroke Foundation Centre for Stroke Recovery, Sunnybrook Health Sciences CentreToronto, Ontario, Canada

**Keywords:** Cognitive impairment, cognitive training, visuospatial/visuomotor

## Abstract

**Introduction:**

Research in the field of the aging brain has evolved to the extent that it is now commonly understood that actively engaging in cognitive tasks provides the potential of being beneficial in affecting the trajectory of age-related cognitive decline. What remains to be examined is the extent, and type, of program required to effect change in aging cognitively impaired individuals.

**Methods:**

To address this issue, a cognitive program focusing on the use of visuospatial (VS)/visuomotor (VM) elements was applied to a group of six older individuals with identified progressive cognitive impairments. It was hypothesized that using tasks with VS and VM components may be beneficial in supporting overall brain performance, and subsequently assist individuals to perform well in various cognitive and behavioral tasks.

**Results:**

Results showed that on many evaluative measures individuals remained stable, or improved in performance with medium-to-large effect sizes (e.g., 0.3–1.0). Thus, in a cognitively impaired population sample where decline would be the norm, our participants improved or remained stable.

**Conclusion:**

The novel application of a VS/VM training program shows promise in addressing global cognitive decline, by targeting a brain area susceptible to early disruptions and providing it with additional and ongoing stimulative tasks in an effort to bolster its functioning and subsequently overall brain functioning.

## Introduction

It is often noted that cognitive decline is one of the primary elements concerning the aging population. In recent years, researchers have focused on nonpharmacological interventions as a way to alleviate deficits associated with cognitive decline. One of the most recent nonpharmacological interventions applied to individuals experiencing cognitive decline or cognitive impairment is a program of cognitive training (CT) (Zanetti et al. 1995; Loewenstein et al. 2004; Sitzer et al. 2006; Acevedo and Loewenstein 2007). Today, this type of procedure is used with individuals from varying populations; however, more historically this type of procedure was used with individuals experiencing traumatic brain injuries (TBI) and since has evolved more recently to be applied to individual's experiencing cognitive declines related to illnesses such as mild cognitive impairment (MCI) as well as individuals with more serious neurodegenerative afflictions, such as Alzheimer's disease (AD) (Loewenstein et al. 2004; Cicerone et al. 2005; Cipriani et al. 2006; Farina et al. 2006; Sitzer et al. 2006).

CT programs vary in their approach of application; the first strategies utilized with individuals experiencing cognitive decline are often ones that are compensatory in nature. Compensatory strategies, for example, involve developing plans to help individuals meet everyday requirements, such as remembering appointments, individual's names, or basic self-grooming skills. Thus, compensatory procedures focus on identifying key areas of deficit and developing strategies and utilizing tools to remediate these deficits. This is in contrast to the restorative approach, where the focus is on holistic remediation through generalized stimulation of the brain (Sitzer et al. 2006). Restorative strategies, therefore, focus on creating a program of general cognitive stimulation (e.g., problem solving and creative activities) aimed at engaging the participant in various tasks that are designed to activate the “brain” generally, and as such, there are no particular tasks tailored to a participant's specified deficit. Both CT approaches have been examined via meta-analytic review, which demonstrated that restorative strategies were noted to be more effective (Sitzer et al. 2006). Specifically, the suggestion is that restorative programs offer significant benefits resulting in the greatest amount of change at posttraining evaluations in both cognitive and functional tasks (Sitzer et al. 2006).

Previously, it has been noted that a reduction in one's visuospatial (VS)/visuomotor (VM) ability can be an early identifier for the onset of a cognitive impairment such as AD (Tales et al. 2002; Tippett and Sergio 2006). Additionally, it can be shown that the brain is highly interconnected with identified bundles of nerve fibers, such as the inferior longitudinal fasciculus, inferior occipitofrontal fasciculus, and posterior thalamic radiation (Voineskos et al. 2012) tying together brain regions such as parietal, an area essential for VS/VM activities (Tippett and Black 2008), frontal, and temporal lobes. Research indicates that damage to these projections noted to support overall brain processing can affect corticocortical projections, and thus could subsequently affect overall performance ability (Behrens et al. 2003; Caeyenberghs et al. 2010; Voineskos et al. 2012). Furthermore, these initial reductions in cognitive ability can be linked to initial deficits observed in one's VM control and VS ability (Johnson et al. 2009). Thus, reductions (a weakened brain area) in one brain region can affect the “support” provided to other brain regions, reducing overall cognitive ability.

However, what can be surmised from current research is that the duration of a program is important, as well as the type of training to be undertaken. For example, above it was noted that restorative approaches appear to be important, but an understanding needs to be achieved into the kind of restorative approaches one should be engaging in. Interestingly, one of the restorative activities identified to be highly beneficial for participants experiencing cognitive decline (i.e., AD) are activities that include VM training (Loewenstein et al. 2004; Acevedo and Loewenstein 2007). In fact, researchers demonstrated that individuals engaging in combination CT programs had the greatest improvements when psychomotor training was present, to the extent that reductions in dementia-related symptoms primarily were seen with only this extra training (Oswald et al. 1996). Additionally, researchers have shown that engaging in tasks such as video game training (VM/VS tasks) results in improvements beyond just VS skills, but improvements could be seen in executive functions (e.g., task switching, working memory, visual short-term memory, and reasoning; Basak et al. 2008; Lee et al. 2012). In addition, it has been suggested by researchers that there is a “interaction of large-scale brain networks when learning with different training strategies” (Voss et al. 2012); thus, our program has included tasks that are ever changing to facilitate novel aspects to create many learning opportunities for the participants. As mentioned above, duration of a program is also essential. In fact, the video gaming studies just described included training times of 23.5 and 20 h (Basak et al. 2008; Voss et al. 2012). Furthermore, a meta-analysis examining CT programs for individuals experiencing AD reported only 19 valid studies (with the exception of 1-year-long study) having an average duration of training of 8.4 weeks (*N* = 18). Thus, with this knowledge we formed a good rationale for the duration of training that we believed would be beneficial to show a positive effect (Sitzer et al. 2006), a 14-week program.

Understanding the role of VS/VM as a training tool, rather than a measure for the presence of an early cognitive impairment, can be an effective way to determine if a specified task, or set of tasks, could be key factors to combating reductions in functional and cognitive ability in a population with recognized difficulties (e.g., AD). There is still a limited amount of evidence available with regards to the rates of success for CT programs for individuals experiencing cognitive decline as a result of age-related diseases. To my knowledge, no CT programs have examined the specified role of VS/VM training in affecting generalized cognitive and functional ability, despite it being currently recognized as a significant factor early in the course of cognitive impairments related to cases of AD (Johnson et al. 2009; McKhann et al. 2011).

The goal of this pilot program was to examine the effects of CT that focus on using mainly VS/VM tasks as a tool to effect positive changes in cognition and functional ability in individuals experiencing cognitive impairments. A program was developed and designed to address current issues (e.g., duration, regular application, and difficulty) and to use mainly VS/VM elements, and thus hypothesized to provide sufficient stimulation to halt and/or positively alter overall cognitive performance. Theoretically, we are postulating that if the brain regions (e.g., posterior partial cortex) supporting VS/VM are affected early in the course of an illness like AD (causing initial cognitive impairments), then providing a program of stimulation, not remediation, could serve to protect this area from further decline and subsequently be available to support other brain regions as cognitive issues arise.

Although internal changes in affected brain structure are suggested, the role of this research is in understanding cognitive/behavioral changes that can be altered via the CT program, and not in measuring structural/anatomical brain changes that might be observable with the application of such a program. However, understanding the full extent of what VS/VM CT can achieve with regards to overall “brain change” will be a topic for further examination.

## Material and Methods

### Participants

Ten participants were recruited from clinical practices, the local hospital's geriatric outpatient clinic, and through community advertisements. Inclusion/exclusion criteria required participants to be between 50 and 85 years of age, have an Mini-Mental State Exam (MMSE) score between 20 and 26, experience an identified cognitive impairment, and to either be on a stable dose of cholinesterase inhibitors (C.I.) for a minimum of 3 months and/or have no medicative intervention to date (all but one participant was on a stable dose of C.I. at least for 3 months prior to study enrollment). Access to medical history was provided by all participants and any participant with known probable secondary causes of dementia or significant comorbidities, neurological, or psychiatric diseases were excluded.

Demographics for participants are presented in Table 1.

**Table 1:** Demographics.

Participants (*N* = 6)	Age	Years of education	Gender	Pre-MMSE
#1	74	9	F	24
#2	57	14	M	24
#3	75	14	F	22
#5	67	16	M	25
#7	77	9	M	21
#10	74	12	M	20
Average	70.6 ± 7.5	12.3 ± 2.9		22.7 ± 0.8

### Procedure

All participants underwent a comprehensive neuropsychological evaluation at baseline and at follow-up (within 30 days following completion of the cognitive stimulation training program). All neuropsychological testing and CT exercises were performed at the University of Northern British Columbia (UNBC) Brain Research Unit. Informed consent was obtained from individual participants as well as their designated caregiver. All procedures and the collection of data were in accordance with UNBC Research Ethics Board's policy.

### Neuropsychological testing

The neuropsychological test battery (Table 2) took less than 90 min to complete. It was constructed to include all major cognitive domains, be a reasonable time period to administer, and ensure a sufficient range of difficulty (floor and ceiling effects) for participants experiencing cognitive impairments. In addition, the Neuropsychiatric Inventory (Basak et al. 2008) and the disability assessment for dementia (Cherrier et al. 2001) were also included to provide indices for functions of ability.

**Table 2:** Neuropsychological test administered.

Global cognition
Mini-Mental State Examination
Mattis Dementia Rating Scale (DRS)
Specific cognitive domains
California Verbal Learning Test
WMS-R Forward Digit Span, WMS-R Backward Digit Span
WMS-R Immediate Visual Reproduction
FAS Fluency, trails A and B
Boston Naming Test
Benton Judgment of Line Orientation, Rey–Osterrieth Complex Figure (Rey-O) Copy and Delay
Western Aphasia Battery-Apraxia subtest
Behavior and ADLs
The Neuropsychiatric Inventory
Disability Assessment in Dementia

### Cognitive stimulation training

Sessions occurred four times per week over a 14-week period, and consisted of approximately two weekly 2-h on-site sessions and two weekly 1-h in-home sessions. On-site testing at UNBC included: (1) navigation task: participants were provided a standard map of the University and asked to find and return from a marked location on the map. Locations were standardized so each participant went to the same location during the specified training session. (2) Visuomotor training: (a) a commercially available “Plug and Play” “Pac-Man” game was used for VM training. The rationale for using Pac-Man is that it requires substantial visually guided ability/control to navigate a virtual environment and it automatically increases in difficulty as the individual improves in ability. (3) Visuoconstruction procedures: this training required participants to complete three tasks. These tasks included variations in: block design (WAIS-IV), a correct fold task (Abner et al. 2012), and a mental rotation task (Shepard and Metzler 1988). In-home sessions consisted of workbook activities comprising VS, visuoconstructive, and VM tasks (e.g., “find the differences,” “correct fold tasks,” “mazes,” etc.). Participants were encouraged to engage in these tasks for a minimum of two weekly sessions of 1 h each week for the duration of the study; 100% compliance was achieved, as measured through weekly tracking sheets.

### Data analysis

#### Neuropsychological tests

Neuropsychological analysis was used to characterize participant's level of cognitive ability at both pre- and posttraining sessions. The role of this analysis is to track changes in cognitive performance in a population that we would expect to have a progressive decline in overall cognition (Zanetti et al. 1995). Thus, a reduction in cognitive performance would be expected to be observed particularly when the population is tracked over a course of 4–5 months.

#### Training tasks

The training tasks were the (cognitive) tools postulated to provide stimulation for the participant's brain. Although the role of these tasks was to create positive cognitive change as measured via neuropsychological tests, these tasks, in and of themselves, are informative about the participant's abilities and cognitive progress. Typically, individuals experiencing significant cognitive difficulties are thought not to possess a great capacity for novel learning (Cherrier et al. 2001). As such, the expectation would be that individuals would struggle to complete these training tasks and show limited progress. To provide a characterization of effects of training performance, and to determine whether individuals were learning these procedures, participants' first 3 weeks of training versus the last 3 weeks were analyzed.

## Results

### Participant progress

Ten participants were originally recruited to participate; however, one declined (#4) to continue the entire 14-week program after week 5 citing transportation concerns (MMSE was 26 well within range of other participants). Participants #8 and #9 showed for initial interview and consent process, but did not show for their baseline neuropsychological assessment (no reason provided). Participant #6 received a comorbid diagnosis (another neurodegenerative condition) while training and was subsequently excluded from the remainder of the program and their data discarded.

### Neuropsychological results

**Figure 1:**
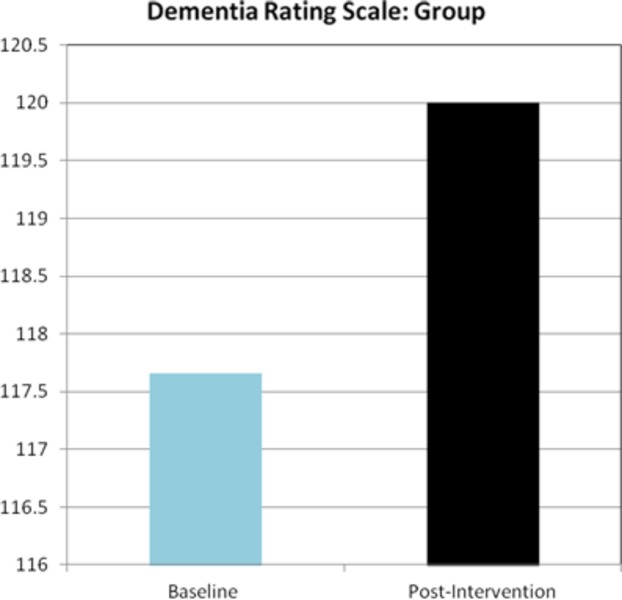
Dementia rating scale DRS demonstrating raw score values at baseline versus postevaluation.

At the completion of the training program, a selection of the most commonly used and well-validated neuropsychological tests demonstrated that participants showed fairly stable performance when pretraining results were compared with posttraining results. Paired samples *t*-test conducted on Dementia Rating Scale (DRS), *t*(5) = −1.03, *P* = 0.346; MMSE, *t*(5) = −1.45, *P* = 0.210; Boston Naming Test (BNT), *t*(5) = −0.20, *P* = 0.849; Benton Line Orientation (BLO), *t*(5) = −0.645, *P* = 0.547; FAS, *t*(5) = −1.05, *P* = 0.341; visual reproduction (VR)-I, *t*(5) = −1.55, *P* = 0.182; digit span forward, *t*(5) = 0.889, *P* = 0.415; digit span backward, *t*(5) = 0.655, *P* = 0.542; Rey-O copy, *t*(4) = −2.25, *P* = 0.087; Rey-O delay, *t*(5) = −0.598, *P* = 0.576; Trails A, *t*(5) = −0.435, *P* = 0.682; Trails B, *t*(4) = 2.00, *P* = 0.116 revealed no significant differences from pretraining to posttraining values (see Fig. 1 for DRS scores).

However, although significance was not found, it is important to note that both the DRS and MMSE had an overall increase in their raw scores. Also, as shown in Table 3, a medium-to-large effect size value was found on the MMSE measure.

**Table 3:** Neuropsychological tests.

Neuropsychological test	Pretraining (mean ± SEM)	Posttraining (mean ± SEM)	Effect size values Cohen's (*d*)
Dementia rating scale (DRS)	117.66 ± 5.5	120 ± 6.7	0.15
Mini-Mental State Exam (MMSE)	22.7 ± 0.8	24.7 ± 1.6	0.61
Boston Naming Test (BNT)	25 ± 2.0	25.2 ± 2.4	0.03
Benton line orientation (BLO)*	22 ± 1.6	23 ± 1.7	0.21
FAS	10.33 ± 1.3	9.5 ± 1.1	0.30
CVLT (A)*	29.2 ± 5.8	34 ± 6.2	1.0
WMS-R visual reproduction (VR-I)	17.66 ± 2.3	23.33 ± 3.1	0.84
WMS-R forward digit span (DS-F)	7.50 ± 0.95	7.0 ± 1.1	0.20
WMS-R backward digit span (DS-B)	5.00 ± 0.81	4.50 ± 0.56	0.29
Rey-O, copy*	26.80 ± 3.5	29.1 ± 2.9	0.32
Rey-O, delay	2.67 ± 1.8	3.17 ± 1.5	0.12
Trail A	78.16 ± 33.9	82.50 ± 32.1	0.13
Trail B*	158.60 ± 33.5	143.80 ± 40.2	0.17

* *N* = 5.

#### Neuropsychological testing results

However, california verbal learning test (CVLT) (acquisition) on follow-up did show a significant improvement from pre- to postanalysis *t*(4) = −12.82, *P* < 0.001.

### Training task results

#### Navigation and block design training

**Figure 2:**
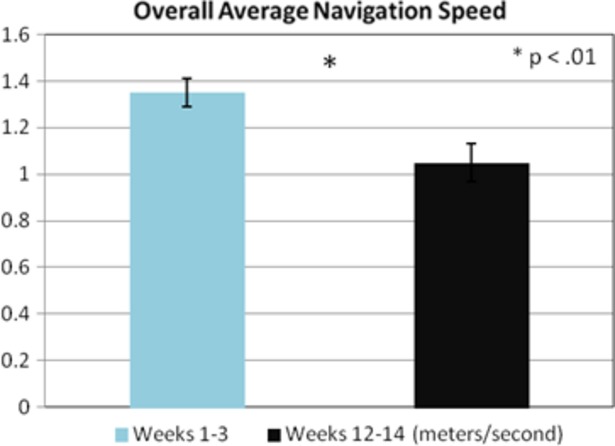
An overall significant group difference was found between weeks 1–3 versus weeks 12–14 on average navigation speed (*P* < 0.01). Effect size value was 0.68 (Cohen's *d*).

**Figure 3:**
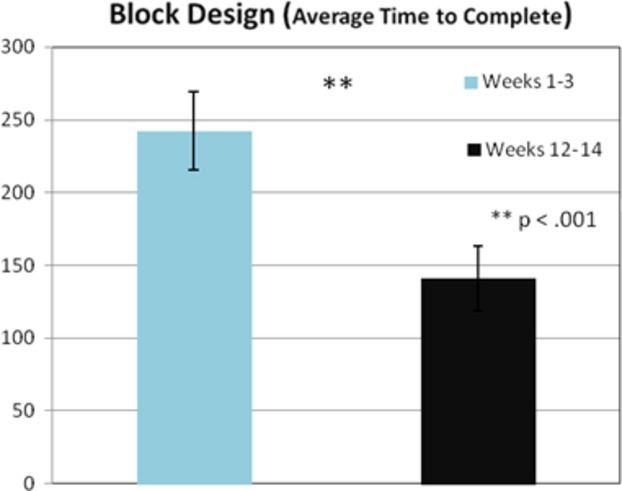
An overall significant group difference was found on block design between weeks 1–3 versus weeks 12–14 (*P* < 0.001). Effect size value was 0.61 (Cohen's *d*).

Paired *t*-tests were utilized to examine participant's ability to both navigate their way around their environment and on their performance for the block design task. Overall results indicated that participants performed significantly better in the last 3 weeks of training than the first 3 weeks of training: navigation speed *t*(26) = 3.39, *P* < 0.01 and block design (completion speed) *t*(33) = 4.98, *P* < 0.001 (see Figs. 2, 3).

#### Game training

**Figure 4:**
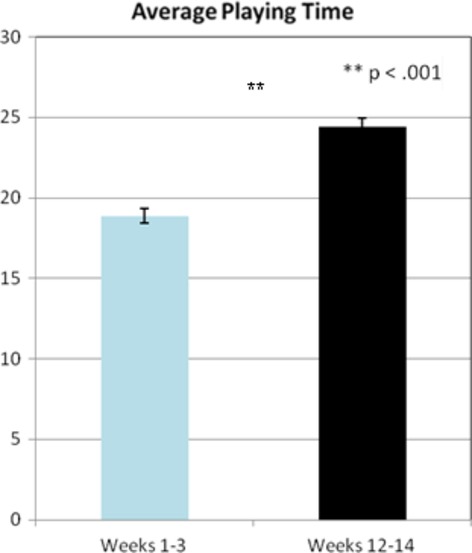
Average game time play was combined for all participants for their first 3 weeks versus their last 3 weeks of training is displayed (*P* < 0.001).

**Figure 5:**
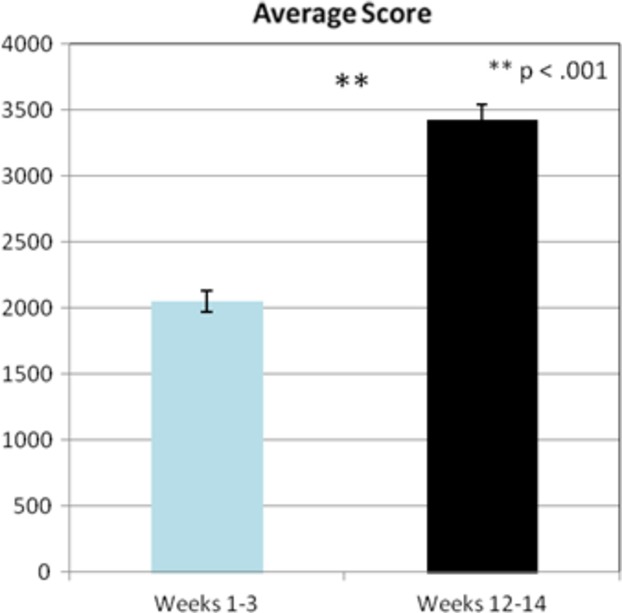
Displayed are the combined values comparing average scores measured for the first 3 weeks versus the last 3 weeks of training (*P* < 0.001).

All participants were tracked each session on their ability to play the game “Pac-Man.” Tracking included duration of play and score achieved. Univariate analysis displayed significant differences in duration of play, *F*_1,1390_ = 70.89; *P* < 0.001, and in cumulative score achieved, F_1,455_ = 140.01; *P* < 0.001. These results indicate that participants had substantial improvement in their ability to play the game longer and to achieve higher scores (see Figs. 4, 5).

#### Behavior changes

**Figure 6:**
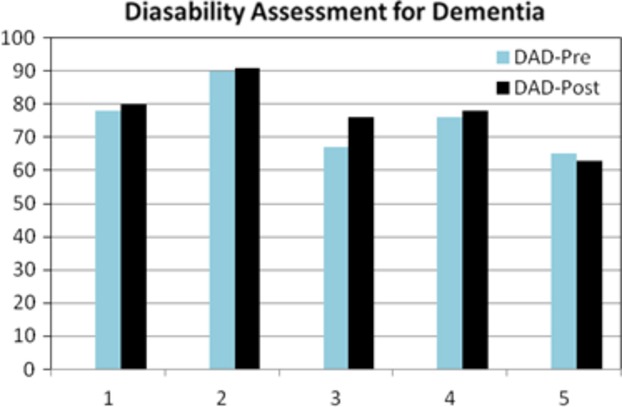
Participant individual results for disability assessment for dementia (DAD) measures. Figure shows that with the exception of one participant all individuals displayed gains at the conclusion of CT.

To examine the effects of behavioral changes for each participant at pre and posttraining time points, the disability for dementia (DAD) and the Western Aphasia Battery-Apraxia (WAB-A) tests were administered. Results demonstrate that no significant differences were observed: WAB-A *t*(5) = −1.34, *P* = 0.237 and DAD *t*(4) = −1.32, *P* = 0.255. However, the overall DAD score increased by 2.4 percentage points and the WAB-A score increased by 6.3 points from pre- to posttraining (see Fig. 6 for DAD scores).

## Discussion

The novel approach of this study was utilizing VS/VM activities as tools for the remediation of cognitive and functional ability in a population with demonstrated cognitive impairments. First and foremost, it is important to note that cognitive improvement or a stabilization in cognitive ability (which can be viewed as improvement) was observed in a population more likely to experience a decline. Improvements on tests of global cognitive ability as seen through increases in overall scores for both the MMSE and DRS were found, as well as a medium effect size was achieved on the MMSE. In addition, a significant pre- to postmeasure difference was found on the CVLT measures with a large accompany effect size, suggesting that overall cognitive performance was being positively affected. Again, although only a raw score increase could be found with regards to the remaining measures, it is important to note that raw score increases are being observed in a population thought to most likely demonstrate a steady cognitive decline. Specifically, it is clinically noted that on average each year a decline in MMSE of 1.8–4.2 points can be expected in, for example, AD populations; thus, a similar decline would be expected for these participants (Zanetti et al. 1995) rather than demonstrating gains. Although *P*-value significance cannot be observed on global cognitive results such as MMSE, there was a clear increase with a low-to-moderate effect size value. Thus, the lack of *P*-value significance needs to be tempered by the observation that declines are not occurring and a nonsignificant result (statistically) would suggest a cognitive gain.

Although with the additional five subjects added to DRS and additional four subjects added to the MMSE (based on average of 2-point increase from pre to post), it is estimated that results would reach statistical significance (*P* < 0.05) for both of these global measures and certainly merit additional research be undertaken. Thus, the next phase for this research is in creating a controlled clinical trial using the training outlined here, which will include both an experimental group of 20 participants (large enough as noted above to show significance), and a controlled group (20 participants) that will be engaged in one type of training (e.g., trivia questions). The goal will be to demonstrate that novel learning and VS and VM focused activities are essential for combating cognitive decline.

CT results for populations experiencing cognitive impairment have been mixed to date. However, as noted earlier, the restorative approaches appear to be the most beneficial, indicating that the primary way to create better cognitive performance is through a program of generalized brain stimulation rather than specified compensatory activities (Sitzer et al. 2006).

The training programs utilized in this research were successful in their ability to create a situation where learning took place and the overall ability of participants increased on all tasks through successive weeks. Interestingly, research to date has noted that adult neurogenesis occurs and is linked to an adult's learning and memory processes (Gould et al. 1999; Deng et al. 2010). Thus, as participants demonstrate the ability to learn and increase their performance throughout training sessions, one might suggest that neuronal growth was achieved and new connections were developed to handle this new information. This is remarkable, as this is a population experiencing dementia-related cognitive impairments, and thus, the expectation is a steady cognitive decline rather than increases in brain mechanisms to support learning. Although in vivo evidence is not presented for this conclusion, the results of the present pilot trial advocate for follow-up with an experiment including the collection of such information.

Current research has shown that elderly individuals transitioning from healthy aging to, for example, AD (sample size of 444) demonstrated a consistent and interesting trait, in that VS measures decline faster than any other cognitive abilities Johnson et al. 2009. In fact, these researchers showed that a decline in VS ability was observed on average to occur as early as 1 year prior to any other cognitive measures demonstrating a similar trend (Johnson et al. 2009). Therefore, with the knowledge that (1) restorative approach appears to be most beneficial to helping individuals with cognitive impairments and (2) that VS ability declines early in this process, application of this pilot program was developed based on its novelty and approach in trying to address generalized cognitive concerns with the stimulation of a specified brain region(s). Brain region(s) supporting VS/VM ability therefore might be the impetus for causing a cascading effect of reduced overall ability as a region of support for other brain areas affected by the progression of cognitive impairments. Rather than trying to implicitly understand the underlying brain process involved (which was well out of the scope of the program), the goal was in trying to understand the role of this CT intervention in effectively combating the cognitive and behavioral systems related to identified cognitive impairments. Although it is still unmistakable that declines are inevitable in populations such as AD afflicted individuals, theories such as cognitive reserve (Sole-Padulles et al. 2009; Bosch et al. 2010) and CT (Loewenstein et al. 2004) certainly suggest that individuals have options for reducing the cognitive and behavioral effects associated with illnesses such as these. In addition, as reversing or stabilizing systems of dementia-related illnesses are still genetically unattainable, it is important to identify ways to combat these impairments and find the “best practice” strategies on how to alter the progressive effects.

### Limitations

One of the limitations of this study could be identified as the number of available patients. However, a meta-analysis of CT tasks for individuals experiencing cognitive impairments indicates that average sample size for this type of research is approximately 16 with the range starting at 7; thus, the number of patients analyzed here is typical of this type of research (Sitzer et al. 2006), as such, although it is a drawback, it certainly is not extraordinary. Additionally, ensuring participants are able to attend each training session and maintain attendance throughout the program was another primary limitation of this research. Future research will have to consider ways to ensure full program completion for all participants. Additionally, whether a 4–5 months period is enough time to determine decline in this population and that improvement is not due to practice effects could be an area of concern. However, as noted earlier we contend that a population such as this should show a yearly average decline in MMSE of 1.8–4.2 points, and over a 4–5 months period one would therefore expect to observe a partial decline in cognition and not general improvements, as was observed in this research. In addition, evidence indicates that practice effects are rare particularly in populations experiencing cognitive impairment. Research has shown that retesting at 1 week showed no effect on tasks such as verbal fluency; thus, at 4–5 months we can feel fairly secure that what is seen is not a practice effect, particularly on these types of tasks (Cooper et al. 2010). As well, evidence suggests that individuals with cognitive impairments given only one follow-up test should be safe from practice effects (Abner et al. 2012). Thus, we believe these points should adequately address any concerns related to the issue of practice effects.

## Conclusion

The proposed next program phase will be undertaken in a larger center to help facilitate greater number of participants and to ensure an ease in access to the program. The end goal, however, is to demonstrate the success of this program and to develop an in-home system for those individuals who may suffer from lack of access to additional care, such as for individuals even in this pilot program that showed difficulty completing all elements. Providing proper tools and greater access to care in the most efficient and effective manner is our ultimate goal.
